# Cancer-Related Fatigue After Esophageal Cancer Surgery: Impact of Postoperative Complications

**DOI:** 10.1245/s10434-021-11049-z

**Published:** 2021-11-23

**Authors:** Zhao Cheng, Asif Johar, Magnus Nilsson, Pernilla Lagergren

**Affiliations:** 1 Surgical Care Science, Department of Molecular Medicine and Surgery, Karolinska Institutet, Karolinska University Hospital, Stockholm, Sweden; 2 Division of Surgery, Department of Clinical Science, Intervention and Technology (CLINTEC), Karolinska Institutet, Stockholm, Sweden; 3 Department of Upper Abdominal Diseases, Karolinska University Hospital, Stockholm, Sweden; 4 Department of Surgery and Cancer, Imperial College London, London, UK

## Abstract

**Background:**

The impact of postoperative complications on cancer-related fatigue is unknown. This nationwide prospective cohort study aimed to assess the trajectory of cancer-related fatigue and the influence of predefined postoperative complications on cancer-related fatigue up to 2 years after esophageal cancer surgery.

**Methods:**

The patients in this study underwent esophagectomy between 2013 and 2019 in Sweden. The exposure was predefined postoperative complications. The outcome was cancer-related fatigue measured by the fatigue scale of the European Organization for Research and Treatment of Cancer Quality of Life Questionnaire Core 30 (EORTC QLQ-C30) and the EORTC QLQ-Fatigue 12 (QLQ-FA12) questionnaire. Linear mixed-effects models provided adjusted fatigue scores and mean score differences (MDs) with 95% confidence intervals (CIs) between patients with and without predefined complications.

**Results:**

The study enrolled 331 patients. The QLQ-C30 fatigue score increased with clinical relevance among patients with any complications (MD, 5.8; 95% CI, 2.6–9.0) who had a higher Clavien-Dindo classification (grades 2 to 3a: MD, 7.3; 95% CI, 3.1–11.5), a medical complication (MD, 6.9; 95% CI, 3.0–10.7), or a pulmonary complication (MD, 6.9; 95% CI, 2.1–11.6) for 1–1.5 years and remained stable until 2 years after esophagectomy. Similar patterns were found in the QLQ-FA12 fatigue and QLQ-FA12 physical and emotional subscales, but not in the cognitive subscales.

**Conclusions:**

Complications in general and medical and pulmonary complications in particular might be associated with increased cancer-related fatigue after esophagectomy.

**Supplementary Information:**

The online version contains supplementary material available at 10.1245/s10434-021-11049-z.

Cancer-related fatigue is a frequently reported distressing sense of tiredness related to both cancer and cancer treatment, including surgery-related fatigue, which cannot not be alleviated by rest or sleep.^1,2^ Cancer patients and survivors usually complain about incomplete role and activity involvement due to the lack of energy, and their quality of life is affected in all dimensions throughout their whole survivorship.^3,4^

Cancer-related fatigue is prevalent across different cancer survivors but may vary between cancer types.^5–7^ Besides, the understanding of cancer-related fatigue among specific cancer types, particularly cancer types with poor prognosis and survivorship, still is lacking and warranted.

In 2018, esophageal cancer ranked seventh in cancer incidence and sixth in cancer mortality globally.^8^ Esophagectomy remains the curatively intended treatment, followed by a high risk (> 50%) of postoperative complications,^9,10^ and the 5-year survival is only about 30–50% after the surgery.^11–13^

Recent Swedish cohort studies found that postoperative complications had a negative impact on health-related quality of life after esophagectomy.^14,15^ However, the role of complications in cancer-related fatigue is only roughly reported, and fewer studies have examined specific conditions of complications in cancer-related fatigue. Such knowledge could help identify patient subgroups under increased burden of cancer-related fatigue. Detailed and reliable data about the development of cancer-related fatigue also could deepen the understanding of this disorder and facilitate the development of interventional strategies.

Therefore, this study aimed to measure cancer-related fatigue over time and to explore whether specific postoperative complications are followed by more severe cancer-related fatigue among esophageal cancer survivors.

## Methods

### Study Design

This study was based on an ongoing Swedish nationwide, prospective cohort entitled the Oesophageal Surgery on Cancer Patients–Adaptation and Recovery (OSCAR) study.” The OSCAR includes esophageal cancer survivors in Sweden surgically treated since 1 January 2013 and onward. The project was approved by the Regional Ethical Review in Stockholm Board (diary no. 2013/844-31/1), and the informed consent forms were signed by all the participants.

### Data Source and Data Collection

Patients were identified through collaboration with the pathology departments of all eight hospitals performing esophagectomies in Sweden, and the study enrolled the patients who survived 1 year after esophagectomy and were able to participate in the study.

Seven assessments were performed between 1 and 5 years after esophagectomy. The first assessment was performed 1 year after esophagectomy by a research nurse who visited the patients in their homes to guide them through the computer-based questionnaires for collecting data on patient-reported outcomes including cancer-related fatigue. Thereafter, at 1.5, 2, 2.5, 3 and 4 years postoperatively, patients were contacted by the project coordinator and responded to the written questionnaires sent by post. At the 5-year assessment, the research nurse visited the patients and performed the final interview in their homes. For the purpose of this study, all available data up to and including the 2-year follow-up period were used.

Clinical data from the time of surgery were collected by review of medical records according to a predefined protocol to ensure data consistency, including information on pathologic tumor stage, tumor histologic type, treatment, and postoperative complications. Comorbidity data were extracted from the Patient Registry.^16^ Education information was retrieved from the Longitudinal Integration Database for Health Insurance and Labor Market. Body weight data were collected from the medical records and follow-up measurements. Data linkages of the participants were enabled by the unique individual Swedish personal identity number, a 10-digit number assigned to each Swedish resident.^17^

### Exposure

The study exposure was complications, defined as deviations from the normal postoperative course within 30 days after surgery. The included complications with definitions are descripted in Table 2.

### Outcome

The primary outcome was cancer-related fatigue measured at 1, 1.5 and 2 years after esophagectomy. Two questionnaires developed by the European Organization for Research and Treatment of Cancer (EORTC) were used in this study: the fatigue scale of EORTC Quality of Life Questionnaire Core 30 (EORTC QLQ-C30) and an additional module, the EORTC QLQ-Fatigue 12 (EORTC QLQ-FA12). Both questionnaires are validated and sensitive to change.^18,19^

The QLQ-C30 is a 30-item questionnaire evaluating the quality of life of cancer patients, within which cancer-related fatigue is measured by a three-item scale. The QLQ-FA12 is a multidimensional instrument for the measurement of cancer-related fatigue with physical, cognitive, and emotional subscales in conjunction with the QLQ-C30. Fatigue scores from the questionnaires were transformed into scales of 0–100. Missing data for each item were handled in line with the EORTC scoring manual. Higher scores indicate more cancer-related fatigue.

### Statistical Analysis

Complications were analyzed in three ways: (1) occurrence of any complications (no or yes), (2) Clavien-Dindo classification (0–1, 2–3a, or 3b–4),^20^ and (3) four specific complication groups (no or yes): surgical complication, medical complication, pulmonary complication, and cardiac complication.

To account for the patient fatigue level before cancer diagnosis,^3,5^ the QLQ-C30 fatigue score from our Background Population study cohort, a random sample from the Swedish population, was used to calculate a proxy baseline score. Each patient was matched to 45 individuals, on the average, from the Background Population study cohort by age at surgery (5-year time window), sex, education level, and comorbidities.^21^ In addition, the proxy baseline score was calculated as the mean fatigue score of the matched individuals.

Linear mixed-effects models were used to assess the fatigue scores up to 2 years after esophagectomy, with adjustment for the following confounders: proxy baseline QLQ-C30 fatigue score, age at surgery (continuous variable), sex (male or female), education level (<9, 9–12 or >12 years of formal education), pathologic tumor stage (0–1, 2, or 3–4), neoadjuvant therapy (no or yes), Charlson Comorbidity Index (0, 1, or ≥2), tumor histology (adenocarcinoma or squamous cell carcinoma), and weight change 1 year after the surgery (continuous variable). Additional analyses were conducted with further adjustment for the preoperative weight change (the difference between average weight as an adult and weight at operation).

The model included time as a categorical variable to allow for non-linear trajectories. Two-way interaction between exposure and time was included in the model to test the differences in fatigue trajectories between the patients with and without predefined complications. The fixed effects included all the covariates, time, and the interaction between complication and time. The intercept was estimated as a random effect to allow for variability across patients.

The results are presented as model-derived mean scores and mean score differences (MDs) with 95% confidence intervals (CIs) of cancer-related fatigue. The MDs were estimated in two ways to facilitate interpretation: (1) MDs within predefined complication groups over time with the 1-year measurement as a reference and (2) MDs between predefined complication groups at each time point. On the scale of transformed score, MDs of 5–10 indicated small clinical relevance, MDs of 10–15 indicated medium clinical relevance, and MDs higher than15 indicated large clinical relevance.^22,23^ The mixed-effects model allows for missing measurements within individuals. Thus all available data were used.^24^

A sensitivity analysis was performed, excluding observations from a patient who died within 2 months of the fatigue response to remove the impact of potential cancer recurrence on the outcome. An experienced biostatistician (A.J.) was responsible for the statistical analyses, and SAS 9.4 (SAS Institute, Cary, NC, USA) software was used for all analyses.

## Results

### Patients

Between January 2013 and May 2019, 839 patients underwent esophagectomy for esophageal cancer in Sweden. Among these patients, 204 (24.3%) died within 1 year and 113 (13.5%) could not be reached, leaving 522 eligible patients for inclusion in the study. Of the eligible patients, 174 (33.3%) were too sick or did not want to participate and 17 (3.3%) were excluded due to unavailability of clinical data. Thus, 331 patients (63.4%) were included in the 1-year measurement of the current study. At 1.5 years, 306 of these patients were alive, and 231 (75.5%) completed the questionnaires. At 2 years, 259 were alive, and 182 (70.3%) remained in the cohort.

Most of the covariates were distributed evenly between the patients with and without complications (Table 1). In the total cohort, 211 (63.7%) of the patients had at least one postoperative complication. The five most common complications were pneumonia (*n* = 65, 19.6%), atrial fibrillation (*n* = 57, 17.2%), anastomotic insufficiency (*n* = 54, 16.3%), respiratory insufficiency (*n* = 52, 15.7%), and sepsis (*n* = 34, 10.3%) (Table 2).

**Table 1:** Demographics and clinical characteristics of the 331 patients who underwent esophagectomy for esophageal cancer in Sweden

	Complication *n* (%)	
	No	Yes
Total	120 (36.3)	211 (63.7)
Age at operation		
Mean	66.5 ± 8.0	67.3 ± 8.6
Sex		
Male	14 (11.7)	20 (9.5)
Female	106 (88.3)	191 (90.5)
Education level (years)		
< 9	26 (21.7)	49 (23.2)
9–12	51 (42.5)	83 (39.3)
> 12	29 (24.2)	56 (26.6)
Missing	14 (11.6)	23 (10.9)
Weight loss (kg)		
Mean	8.8 ± 7.8	9.4 ± 8.8
Missing	3 (0.03)	13 (0.06)
Pathologic tumor stage		
0–1	42 (35.0)	69 (32.7)
2	40 (33.3)	68 (32.3)
3–4	38 (31.7)	72 (34.1)
Missing	0 (0.0)	2 (0.9)
Neoadjuvant therapy		
No	99 (82.5)	158 (74.9)
Yes	19 (15.8)	49 (23.2)
Missing	2 (1.7)	4 (1.9)
Tumor histology		
Adenocarcinoma	105 (87.5)	172 (81.5)
Squamous cell carcinoma	15 (12.5)	39 (18.5)
Charlson comorbidity index		
0	60 (50.0)	84 (39.8)
1	38 (31.7)	71 (33.7)
≥ 2	22 (18.3)	56 (26.5)

**Table 2:** Postoperative complications within 30 days after esophagectomy for esophageal cancer for 331 patients

Complication	Definition	*n* (%)
Complications	Occurrence of any complications	
No		120 (36.3)
Yes		211 (63.7)
Clavien-Dindo classification	–	
0–1		129 (39.0)
2–3a		116 (35.0)
3b–4		85 (25.7)
Missing		1 (0.3)
Complication group^a^		
Medical complication	Sepsis, pneumonia, hepatic insufficiency, renal failure, deep venous thrombosis, pulmonary embolism, other embolism, myocardial infarction, atrial fibrillation, cerebral infarction, or respiratory insufficiency	146 (44.1)
Surgical complication	Postoperative bleeding, anastomotic insufficiency, substitute necrosis, thoracic ductus injury, intrathoracic abscess or empyema, intra-abdominal abscess, wound infection, wound dehiscence, ileus, gastric perforation, recurrent laryngeal nerve paralysis, or strictures in anastomosis	112 (33.8)
Pulmonary complication	Respiratory insufficiency or pneumonia	97 (29.3)
Cardiac complication	Myocardial infarction or atrial fibrillation	61 (18.4)
Complications type^a^		
Pneumonia	Radiologically detected infiltrate with clinical symptoms such as fever, cough, or dyspnea	65 (19.6)
Atrial fibrillation	Newly electrocardiogram detected and treatment required	57 (17.2)
Anastomotic insufficiency	Clinically significant or radiologically detected	54 (16.3)
Respiratory insufficiency	Reintubation or mechanical ventilation needed	52 (15.7)
Sepsis	Causing clinical symptoms such as fever, chills, and proven bacteria in the blood	34 (10.3)
Wound infection	Causing clinical symptoms and requiring treatment	25 (7.6)
Intrathoracic abscess or empyema	≥3*3 cm radiologically or surgically detected abscess with clinical symptoms such as fever, pain or dyspnea	22 (6.6)
Recurrent laryngeal nerve paralysis	Laryngeal inspection ascertained	20 (6.0)
Pulmonary embolism	Radiologically detected	15 (4.5)
Thoracic ductus injury	Thoracic lymph leakage requiring drainage for more than 7 days or reoperation	15 (4.5)
Substitute necrosis	Clinically significant ischemia with ulceration or perforation	11 (3.3)
Intra-abdominal abscess	≥3*3cm radiologically or surgically detected abscess with clinical symptoms such as fever or pain	7 (2.1)
Myocardial infarction	Electrocardiogram or cardiac enzymes verified	7 (2.1)
Ileus	Radiologically detected ileus in need of surgery	5 (1.5)
Renal failure	Dialysis needed	4 (1.2)
Postoperative bleeding	>2000 ml or requiring reoperation	2 (0.6)
Other embolism	Radiologically detected and requiring treatment	2 (0.6)
Strictures in anastomosis	Endoscopic intervention required	2 (0.6)
Hepatic insufficiency	Progressive jaundice	1 (0.3)
Gastric perforation	Surgical intervention required	1 (0.3)
Wound dehiscence	Clinically obvious wound rupture	0 (0.0)
Deep venous thrombosis	Radiologically or clinically verified with treatment needs	0 (0.0)
Cerebral infarction/stroke	Radiologically verified	0 (0.0)
Other complications^b^	–	38 (11.5)

^a^Each patient could have more than 1 group or type of complications

^b^Most of the patients who had other complications also had the specific complication types listed above

### Fatigue Scores Within Predefined Complication Groups Over Time Points

**Fig. 1:**
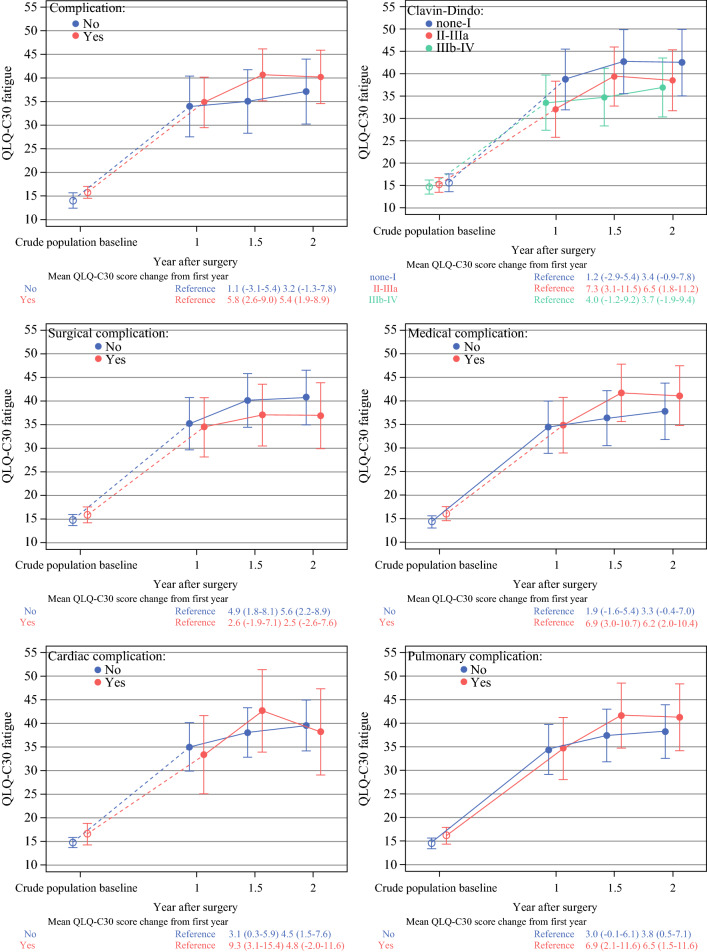
QLQ-C30 fatigue trajectories and mean score differences with 95% confidence intervals between time points by predefined complications

Over time, the trajectory of QLQ-C30 fatigue showed a clinically relevant increase among the patients with complications (MD, 5.8; 95% CI, 2.6–9.0), a higher Clavien-Dindo classification (grades 2–3a: MD, 7.3; 95% CI, 3.1–11.5), a medical complication (MD, 6.9; 95% CI, 3.0–10.7), or a pulmonary complication (MD, 6.9; 95% CI, 2.1–11.6) in 1–1.5 years, then remained stable until 2 years after esophagectomy. But the developments of QLQ-C30 fatigue were almost identical between the patients with and without surgical or cardiac complication (Fig. 1).

**Fig. 2:**
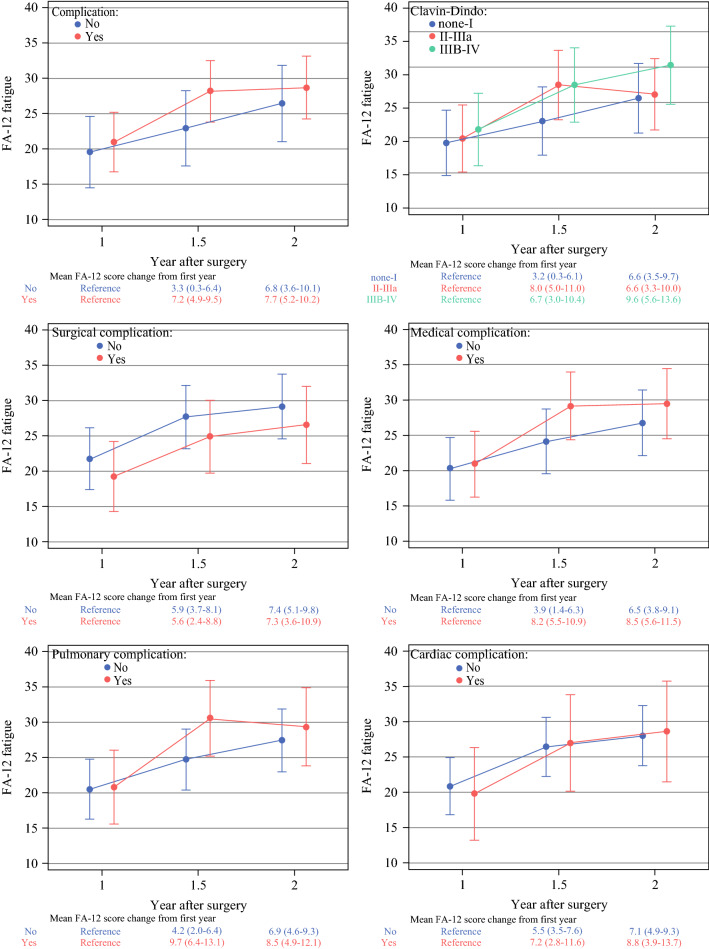
QLQ-FA12 fatigue trajectories and mean score differences with 95% confidence intervals between time points by predefined complication.

Similar to QLQ-C30 fatigue, the QLQ-FA12 fatigue deteriorated for the patients with complications (MD, 7.2; 95% CI, 4.9–9.5), a higher Clavien-Dindo classification (grades 2–3a: MD, 8.0; 95% CI, 5.0–11.0), a medical complication (MD, 8.2; 95% CI, 5.5–10.9), or a pulmonary complication (MD, 9.7; 95% CI, 6.4–13.1) in 1–1.5 years, then remained stable up to 2 years postoperatively. Besides, the QLQ-FA12 fatigue trajectory for the patients with and without pulmonary complications showed different developments over time (*P*_interaction_ = 0.023). Again, the patients with and without surgical or cardiac complications showed similar QLQ-FA12 fatigue trajectories (Fig. 2).

As for the QLQ-FA12 subscales, like the patients with patterns of QLQ-C30 and QLQ-FA12 fatigue, the patients with complications, a higher Clavien-Dindo grade, a medical complication, or a pulmonary complication reported a greater burden of physical and emotional fatigue in 1–1.5 years, and the burden remained until 2 years postoperatively. However, no clinically relevant trajectory differences were found for cognitive fatigue (Figs. A1–A3).

### Fatigue Scores Between Predefined Complication Groups at Each Time Point

Clinically relevant fatigue score differences between the patients with and without predefined complications were found 1.5 years postoperatively. The patients with at least one complication had a higher QLQ-FA12 fatigue score with clinical relevance (MD, 5.3; 95% CI, 0.4–10.2) than those without complications 1.5 years postoperatively. However, for QLQ-C30 fatigue, the difference was clinically relevant but did not reach the level of statistical significance (MD, 5.6; 95% CI, − 0.7 to 12.0). More QLQ-C30 fatigue was found among the patients with a Clavien-Dindo classification grade higher than 3b (MD, 8.0; 95% CI, 0.1–15.8) compared with grades 0–1 at 1.5 years. Besides, the QLQ-FA12 fatigue score increased for the patients with a medical complication (MD, 5.0; 95% CI, 0.2–9.7), notably for those with a pulmonary complication (MD, 5.8; 95% CI, 0.7–11.0), at the 1.5-year follow-up evaluation. No clinically significant MDs were found regarding surgical or cardiac complications at any time points (Table 3).

**Table 3:** Adjusted mean score differences with 95 % confidence intervals in QLQ-C30 and QLQ-FA12 fatigue scores between patients with and without predefined medical complications after esophagectomy at different time points^a^

	QLQ-C30 fatigue			QLQ-FA12 fatigue		
	1 Year	1.5 Years	2 Years	1 Year	1.5 Years	2 Years
Complications						
No	Reference	Reference	Reference	Reference	Reference	Reference
Yes	1.0 (−4.9 to 6.8)	5.6 (−0.7 to 12.0)	3.1 (−3.6 to 9.8)	1.4 (−3.2 to 6.0)	**5.3 (0.4–10.2)**	2.2 (−2.9 to 7.4)
Clavien-Dindo classification						
0–1	Reference	Reference	Reference	Reference	Reference	Reference
2–3a	− 1.4 (− 7.9 to 5.1)	4.7 (− 2.3 to 11.6)	1.6 (−5.8 to 9.0)	0.6 (−4.5 to 5.7)	5.4 (−0.0 to 10.8)	0.6 (−5.1 to 6.3)
3b–4	5.3 (−2.0 to 12.5)	**8.0 (0.1–15.8)**	5.6 (−2.7 to 13.8)	2.0 (−3.7 to 7.7)	5.4 (−0.7 to 11.5)	5.0 (−1.4 to 11.4)
*Complication group*						
Surgical complication						
No	Reference	Reference	Reference	Reference	Reference	Reference
Yes	−0.7 (−6.8 to 5.3)	−3.1 (−9.7 to 3.5)	−3.8 (−10.9 to 3.2)	−2.5 (−7.3 to 2.3)	−2.8 (−7.9 to 2.3)	−2.6 (−8.1 to 2.8)
Medical complication						
No	Reference	Reference	Reference	Reference	Reference	Reference
Yes	0.4 (−5.3 to 6.1)	5.4 (−0.7 to 11.5)	3.3 (−3.2 to 9.8)	0.7 (−3.8 to 5.1)	**5.0 (0.2–9.7)**	2.8 (−2.2 to 7.8)
Pulmonary complication						
No	Reference	Reference	Reference	Reference	Reference	Reference
Yes	0.3 (−5.9 to 6.5)	4.2 (−2.5 to 10.8)	3.0 (−4.0 to 10.0)	0.3 (−4.6 to 5.2)	**5.8 (0.7–11.0)**	1.9 (−3.5 to 7.3)
Cardiac complication						
No	Reference	Reference	Reference	Reference	Reference	Reference
Yes	−1.6 (−9.3 to 6.1)	4.6 (−3.6 to 12.8)	−1.3 (−10.1 to 7.5)	−1.1 (−7.2 to 5.0)	0.6 (−5.8 to 7.0)	0.6 (−6.2 to 7.4)

^a^Values in bold are both clinically relevant and statistically significant

The MDs of the QLQ-FA12 physical, emotional, and cognitive fatigue subscales at each of the time points showed similar patterns. All the clinically relevant MDs were found 1.5 years postoperatively. The patients who had complications experienced greater physical fatigue (MD, 6.5; 95% CI, 0.2–12.8). As for the Clavien-Dindo classification, the patients with grades higher than 3b had more physical fatigue (MD, 8.0; 95% CI, 0.2–15.7), and the patients with grades 2 to 3a showed greater emotional fatigue (MD, 8.1; 95% CI, 1.4–14.8) and cognitive fatigue (MD, 5.7; 95% CI, 0.7–10.8) in contrast to the patients with a grade lower than 1. Medical complication was associated with increased physical fatigue (MD, 6.5; 95% CI, 0.5–12.5). Pulmonary disease was associated with all three subscales of fatigue, specifically physical (MD, 6.8; 95% CI, 0.2–13.4), emotional (MD, 7.4; 95% CI, 1.1–13.8), and cognitive (MD, 6.1; 95% CI, 1.3–11.0) fatigue (Table S1).

The analyses with further adjustment for preoperative weight change provided similar results (Tables S2 and S3; Figs. A4–A8). The results of the sensitivity analysis, excluding the observations from patients who died within 2 months after the response, were almost the same as those stated earlier (data not shown).

## Discussion

This study indicated a high level of cancer-related fatigue in esophageal cancer survivors after esophagectomy. Postoperative medical and pulmonary complications were associated with an increased level of cancer-related fatigue.

To our knowledge, this is the first prospective and longitudinal study to measure cancer-related fatigue among esophageal cancer survivors. The well-validated questionnaires, the nationwide and population-study based design, and the reliable data source counterbalanced the risk of information bias and ensured the generalizability of the results. The EORTC QLQ-C30 fatigue scale emphasizes the physical aspect of fatigue, whereas the EORCT QLQ-FA12 also covers the emotional and cognitive fatigue properties,^25^ but very few studies have used both questionnaires, hampering the comprehensive understanding of the measurements.

This study provided complete results from the two commonly used questionnaires, thus filling the gap and enhancing the comparability with other studies. However, cognitive fatigue might have been underestimated in this study because severe cognitive fatigue could restrict patients’ willingness or ability to participate in the study, indicating that the lack of association must be interpreted cautiously.

The lack of a baseline fatigue measurement was a weakness in this study, but a proxy baseline score from the matched reference cohort was calculated to mimic the fatigue level before cancer diagnosis and adjusted in the analysis to reduce concerns about the influence from host characteristics. Besides, an unmeasured or residual confounder, such as sarcopenia, was inevitable in this observational study. Moreover, some other postoperative complications, including diaphragmatic herniation and delayed gastric conduit emptying, were not available in the current cohort, which hampered the completeness of the assessment for the exposure. Dotted lines were used due to the lack of baseline measurement for the patients, and the fatigue trajectory between baseline and 1 year after esophagectomy could not be imputed. Another limitation was the potential selection bias caused by the patients who declined to participate due to serious illness and severe fatigue, but this could only dilute the associations and not reverse the results.

The esophageal cancer patients had higher fatigue levels after esophagectomy than the background population. One large longitudinal study also found higher fatigue scores among Hodgkin’s lymphoma patients than among the German reference population,^5^ and studies regarding colorectal, breast, ovarian, and endometrial cancer reported a higher fatigue level than at the baseline before treatment.^26–28^

However, the trajectories of fatigue vary among different cancer patients. Studies analyzing Hodgkin’s lymphoma and breast cancer found that the fatigue scores increased dramatically during cancer therapy, then decreased to the pre-treatment level within 1 year and remained at a stable level.^5,27^ Nevertheless, an English cohort found that cancer-related fatigue also changed sharply during the first year after esophagectomy but kept at a higher level than the baseline before treatment.^29^

In the current study, data within 1 year after surgery were not available, but the fatigue score still increased between 1 and 1.5 years, and did not relieve until up to 2 years after the surgery. The reason might stem from the specific survival issues after esophagectomy. Esophageal cancer survivors experience psychiatric distress, eating difficulty, and physical symptoms of pain, cough, and reflux after the surgery,^30^ which could cause sleep disturbance and nutritional deficits, contributing to the poor survivorship with persisting cancer-related fatigue.^3,4^ This also could be the reason why the 1-year fatigue measurements are similar between patients with and those without predefined complications, considering that numerous strong factors exist during the initial postoperative period. As time passes after the surgery, some symptoms are relieved, the patients also gradually adapt to their new life, and the continuous effect of their complications show up.

In this study, the postoperative complications were grouped as surgical and medical complications. Surgical complications such as bleeding and anastomotic insufficiency are related to the surgical procedure, whereas medical complications usually are medical diseases such as pneumonia and myocardial infarction. Previous studies have found that postoperative complications, especially medical complications, were associated with long-lasting impaired health-related quality of life after esophagectomy.^14,15^

The current study provided further evidence regarding the effect of medical complications on cancer-related fatigue in detail. Patients with medical complications, specifically pulmonary complications, might already have chronic lung disease and worse performance status before the surgery.^31,32^ Such long-term comorbidities and complications that add disease burden^5,26,27^ and activate the inflammatory process and immune system reaction^2,4^ seem to increase the risk and severity of cancer-related fatigue. The effects of medical complications in the current study existed even after the adjustment for comorbidities. The lack of association between cardiac complications and cancer-related fatigue may be explained by the fact that the most common cardiac disorder, atrial fibrillation, was resolved in most cases at discharge and had no impact on long-term survival after esophagectomy.^33,34^ Previous Swedish studies have suggested that surgical complications healed in the long term, and that the influence on the QLQ-C30 fatigue score was diminished 5 years after esophagectomy.^14,35^ However, the current study found a limited effect of surgical complications on cancer-related fatigue 1 to 2 years after the surgery. A possible reason could have been the different calendar periods. The patients in the current cohort were surgically treated after 2013 and might have been relieved of surgical complications sooner due to the advanced surgical techniques and supportive care compared with the patients in the former studies conducted before 2005.

The survival of esophageal cancer patients has been improving, and the way to promote the health-related quality of life after treatment has become an increasing interest for esophageal cancer patients together with the prolonged survival. Cancer-related fatigue is one of the most severe symptoms influencing the quality of life after esophagectomy,^36^ but no acknowledged treatment intervention has been discovered. This study provided evidence that reducing cancer-related fatigue could be achieved by the prevention of postoperative complications. Moreover, the results also emphasized the importance of considering individual effects of different complications. The risk of medical complications may be reduced by careful selection and optimization of patients, particularly pulmonary optimization. Minimally invasive surgery also is credited for the low incidence of pulmonary complications.^37^ As for patients with low pulmonary function before or after the surgery, rehabilitative intervention and long-term follow-up evaluation should be supported.

In summary, this prospective, population-based cohort study showed that medical and pulmonary complications might be associated with an increased level of cancer-related fatigue for esophageal cancer survivors. The trajectory of cancer-related fatigue increased in 1–1.5 years, then remained stable until 2 years after esophagectomy. These findings indicate the need for personalized and long-term follow-up evaluation for patients, accompanied by medical complications and the relevance of considering complications during the rehabilitation, with tailored support to counteract cancer-related fatigue after esophagectomy.

## Supplementary Information

Below is the link to the electronic supplementary material.

Supplementary file1 (PDF 1287 kb)
