# Supplementary material for: Cancer-Related Fatigue After Esophageal Cancer Surgery: Impact of Postoperative Complications
Source: Ann Surg Oncol. 2021 Nov 23;29(5):2842–51. doi: 10.1245/s10434-021-11049-z (PMC8989940; doi:10.1245/s10434-021-11049-z)
Supplement: Supplementary file 1 — Supplementary file1 (PDF 1287 kb) [file 10434_2021_11049_MOESM1_ESM.pdf]

Supplementary material:

Table S1. Adjusted mean score differences with 95% confidence intervals in QLQ-FA12 subscale fatigue scores between patients with and without predefined medical complications after esophagectomy at different time points

	**QLQ-FA12 physical fatigue**			**QLQ-FA12 emotional fatigue**			**QLQ-FA12 cognitive fatigue**		
	**1 year**	**1.5 years**	**2 years**	**1 year**	**1.5 years**	**2 years**	**1 year**	**1.5 years**	**2 years**
Complications									
No	Reference	Reference	Reference	Reference	Reference	Reference	Reference	Reference	Reference
Yes	1.5 (-4.3-7.4)	**6.5 (0.2-12.8)**	3.0 (-3.6-9.6)	1.5 (-4.1-7.1)	5.8 (-0.3-11.9)	2.2 (-4.2-8.6)	2.4 (-1.9-6.6)	4.4 (-0.3-9.0)	1.7 (-3.2-6.7)
Clavien-Dindo classification									
0-I	Reference	Reference	Reference	Reference	Reference	Reference	Reference	Reference	Reference
II-IIIa	-1.4 (-7.8-5.1)	4.5 (-2.4-11.4)	0.6 (-6.8-7.9)	2.3 (-3.9-8.4)	**8.1 (1.4-14.8)**	0.9 (-6.2-8.0)	2.3 (-2.3-7.0)	**5.7 (0.7-10.8)**	0.6 (-4.8-6.1)
IIIb-IV	3.6 (-3.6-10.9)	**8.0 (0.2-15.7)**	4.8 (-3.4-12.9)	1.0 (-5.9-7.9)	4.9 (-2.6-12.4)	6.4 (-1.5-14.3)	3.6 (-1.5-8.8)	4.2 (-1.6-9.9)	4.9 (-1.1-11.0)
Complication group									
Surgical complication									
No	Reference	Reference	Reference	Reference	Reference	Reference	Reference	Reference	Reference
Yes	-3.0 (-9.0-3.1)	-2.1 (-8.6-4.4)	-3.8 (-10.8-3.2)	-1.7 (-7.5-4.1)	-3.2 (-9.5-3.1)	-2.6 (-9.4-4.2)	0.1 (-4.3-4.5)	-2.8 (-7.6-2.0)	0.1 (-5.2-5.3)
Medical complication									
No	Reference	Reference	Reference	Reference	Reference	Reference	Reference	Reference	Reference
Yes	1.1 (-4.5-6.7)	**6.5 (0.5-12.5)**	3.2 (-3.2-9.6)	-0.3 (-5.7-5.2)	5.1 (-0.8-10.9)	3.8 (-2.4-10.0)	1.1 (-3.0-5.2)	3.8 (-0.6-8.2)	1.1 (-3.6-5.9)
Pulmonary complication									
No	Reference	Reference	Reference	Reference	Reference	Reference	Reference	Reference	Reference
Yes	1.2 (-5.0-7.4)	**6.8 (0.2-13.4)**	2.0 (-4.9-9.0)	-1.0 (-6.9-4.9)	**7.4 (1.1-13.8)**	3.6 (-3.1-10.3)	-0.1 (-4.6-4.3)	**6.1 (1.3-11.0)**	-0.2 (-5.3-5.0)
Cardiac complication									
No	Reference	Reference	Reference	Reference	Reference	Reference	Reference	Reference	Reference
Yes	-1.8 (-9.5-5.9)	1.6 (-6.6-9.7)	-0.7 (-9.4-8.1)	-1.0 (-8.3-6.4)	-1.7 (-9.6-6.1)	2.0 (-6.5-10.4)	2.3 (-3.2-7.9)	0.0 (-5.9-6.0)	0.1 (-6.4-6.6)

Values in bold are both clinically relevant and statistically significant.

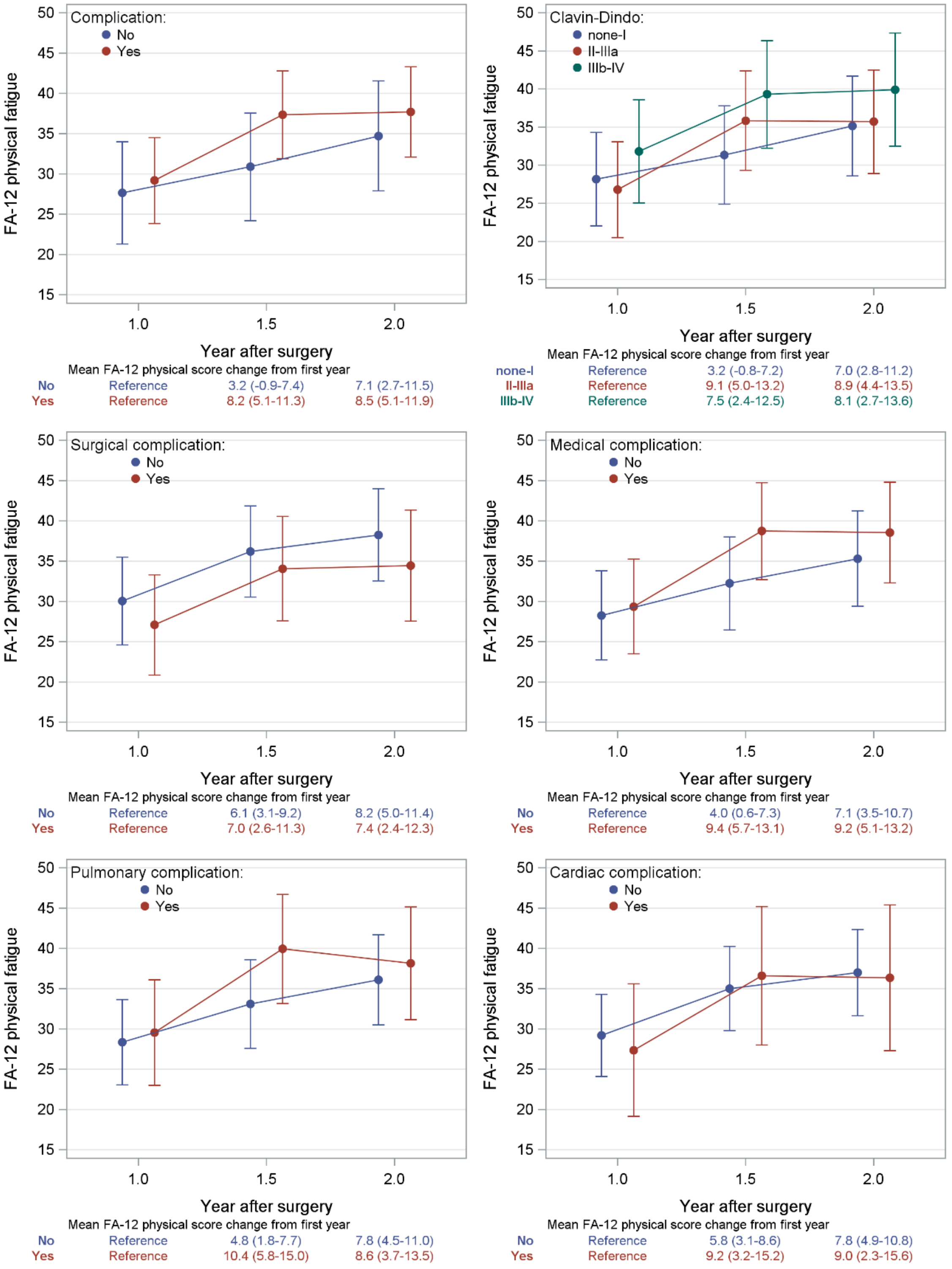

Figure A1. QLQ-FA12 physical fatigue trajectories and mean score differences with 95% confidence intervals between time points by predefined complications

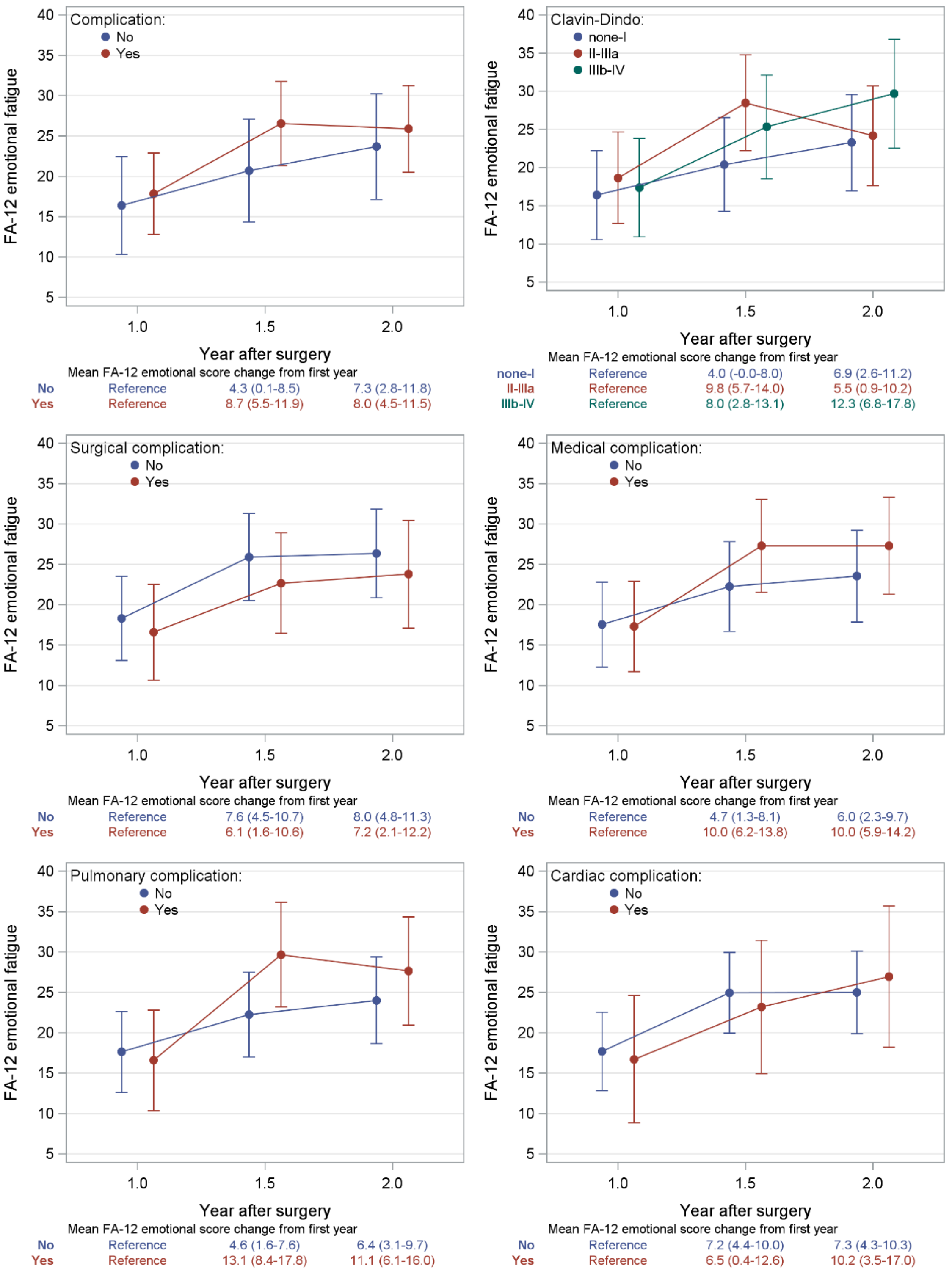

Figure A2. QLQ-FA12 emotional fatigue trajectories and mean score differences with 95% confidence intervals between time points by predefined complications

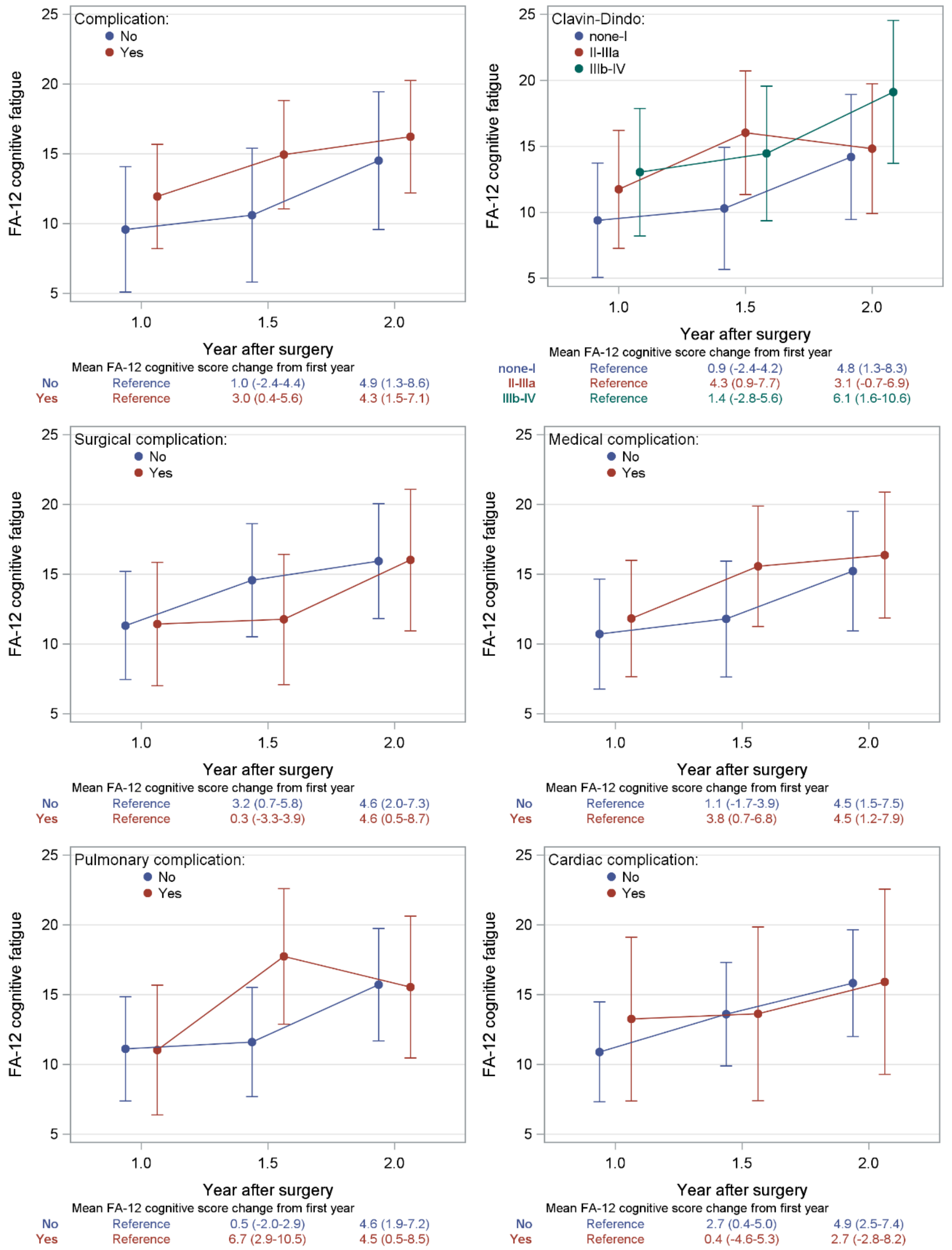

Figure A3. QLQ-FA12 cognitive fatigue trajectories and mean score differences with 95% confidence intervals between time points by predefined complications

Table S2. Adjusted mean score differences with 95% confidence intervals in QLQ-C30 and QLQ-FA12 fatigue scores between patients with and without predefined medical complications after esophagectomy at different time points (further adjusted for preoperative weight change)

	**QLQ-C30 fatigue**			**QLQ-FA12 fatigue**		
	**1 year**	**1.5 years**	**2 years**	**1 year**	**1.5 years**	**2 years**
Complications						
No	Reference	Reference	Reference	Reference	Reference	Reference
Yes	0.5 (-5.6-6.7)	4.8 (-1.8-11.4)	1.7 (-5.2-8.7)	1.8 (-3.1-6.6)	**5.5 (0.3-10.6)**	2.0 (-3.3-7.4)
Clavien-Dindo classification						
0-I	Reference	Reference	Reference	Reference	Reference	Reference
II-IIIa	-1.8 (-8.4-4.9)	4.2 (-3.0-11.3)	1.6 (-6.0-9.1)	1.1 (-4.2-6.4)	5.5 (-0.1-11.1)	0.8 (-5.1-6.8)
IIIb-IV	4.1 (-3.5-11.7)	6.7 (-1.5-14.8)	4.4 (-4.2-13.0)	1.5 (-4.5-7.6)	5.3 (-1.2-11.7)	4.7 (-2.0-11.4)
Complication group						
Surgical complication						
No	Reference	Reference	Reference	Reference	Reference	Reference
Yes	-1.5 (-7.8-4.8)	-4.5 (-11.3-2.2)	-4.3 (-11.5-3.0)	-2.4 (-7.4-2.6)	-3.4 (-8.7-1.9)	-2.9 (-8.5-2.7)
Medical complication						
No	Reference	Reference	Reference	Reference	Reference	Reference
Yes	-0.7 (-6.5-5.2)	4.7 (-1.6-11.0)	2.9 (-3.8-9.6)	0.5 (-4.1-5.2)	**5.3 (0.4-10.2)**	3.0 (-2.2-8.2)
Pulmonary complication						
No	Reference	Reference	Reference	Reference	Reference	Reference
Yes	-0.4 (-6.8-6.0)	3.6 (-3.2-10.5)	2.8 (-4.4-10.0)	0.3 (-4.8-5.4)	**6.4 (1.0-11.8)**	2.2 (-3.4-7.8)
Cardiac complication						
No	Reference	Reference	Reference	Reference	Reference	Reference
Yes	-2.5 (-10.4-5.5)	4.2 (-4.1-12.6)	-2.3 (-11.3-6.7)	-1.8 (-8.1-4.5)	0.6 (-6.0-7.2)	0.5 (-6.5-7.6)

Values in bold are both clinically relevant and statistically significant.

Table S3. Adjusted mean score differences with 95% confidence intervals in QLQ-FA12 subscale fatigue scores between patients with and without predefined medical complications after esophagectomy at different time points (further adjusted for preoperative weight change)

	**QLQ-FA12 physical fatigue**			**QLQ-FA12 emotional fatigue**			**QLQ-FA12 cognitive fatigue**		
	**1 year**	**1.5 years**	**2 years**	**1 year**	**1.5 years**	**2 years**	**1 year**	**1.5 years**	**2 years**
Complications									
No	Reference	Reference	Reference	Reference	Reference	Reference	Reference	Reference	Reference
Yes	1.7 (-4.4-7.9)	**6.8 (0.3-13.3)**	3.0 (-3.9-9.8)	2.4 (-3.5-8.3)	6.4 (0.1-12.8)	2.0 (-4.7-8.7)	2.4 (-2.1-6.8)	3.8 (-1.1-8.6)	1.3 (-3.8-6.5)
Clavien-Dindo classification									
0-I	Reference	Reference	Reference	Reference	Reference	Reference	Reference	Reference	Reference
II-IIIa	-1.0 (-7.7-5.7)	4.7 (-2.4-11.9)	1.2 (-6.4-8.7)	3.1 (-3.3-9.6)	**8.5 (1.5-15.4)**	1.0 (-6.4-8.3)	2.4 (-2.4-7.3)	5.2 (-0.0-10.5)	0.6 (-5.0-6.2)
IIIb-IV	2.7 (-4.9-10.3)	**7.9 (-0.2-16.0)**	4.8 (-3.7-13.4)	1.4 (-5.9-8.7)	5.0 (-2.9-12.9)	5.9 (-2.5-14.2)	3.4 (-2.1-8.9)	3.0 (-3.0-9.0)	4.7 (-1.7-11.0)
Complication group									
Surgical complication									
No	Reference	Reference	Reference	Reference	Reference	Reference	Reference	Reference	Reference
Yes	-2.9 (-9.2-3.4)	-3.0 (-9.7-3.7)	-3.9 (-11.1-3.3)	-1.5 (-7.5-4.6)	-3.4 (-10.0-3.1)	-2.9 (-10.0-4.1)	0.2 (-4.4-4.7)	-3.3 (-8.2-1.7)	0.6 (-4.9-6.0)
Medical complication									
No	Reference	Reference	Reference	Reference	Reference	Reference	Reference	Reference	Reference
Yes	0.8 (-5.1-6.7)	**6.5 (0.3-12.8)**	3.5 (-3.1-10.2)	0.1 (-5.6-5.8)	5.7 (-0.4-11.8)	3.8 (-2.6-10.3)	1.2 (-3.1-5.4)	4.3 (-0.4-8.9)	1.9 (-3.1-6.8)
Pulmonary complication									
No	Reference	Reference	Reference	Reference	Reference	Reference	Reference	Reference	Reference
Yes	1.2 (-5.2-7.6)	**7.2 (0.4-14.0)**	2.6 (-4.6-9.7)	-0.6 (-6.7-5.6)	**8.4 (1.8-15.1)**	3.9 (-3.1-10.8)	-0.1 (-4.8-4.5)	**6.7 (1.7-11.7)**	0.6 (-4.8-5.9)
Cardiac complication									
No	Reference	Reference	Reference	Reference	Reference	Reference	Reference	Reference	Reference
Yes	-3.0 (-10.9-4.9)	1.5 (-6.8-9.9)	-1.0 (-9.9-8.0)	-1.0 (-8.7-6.6)	-1.8 (-9.9-6.3)	1.6 (-7.1-10.4)	2.3 (-3.5-8.0)	0.0 (-6.1-6.2)	0.1 (-6.6-6.8)

Values in bold are both clinically relevant and statistically significant.

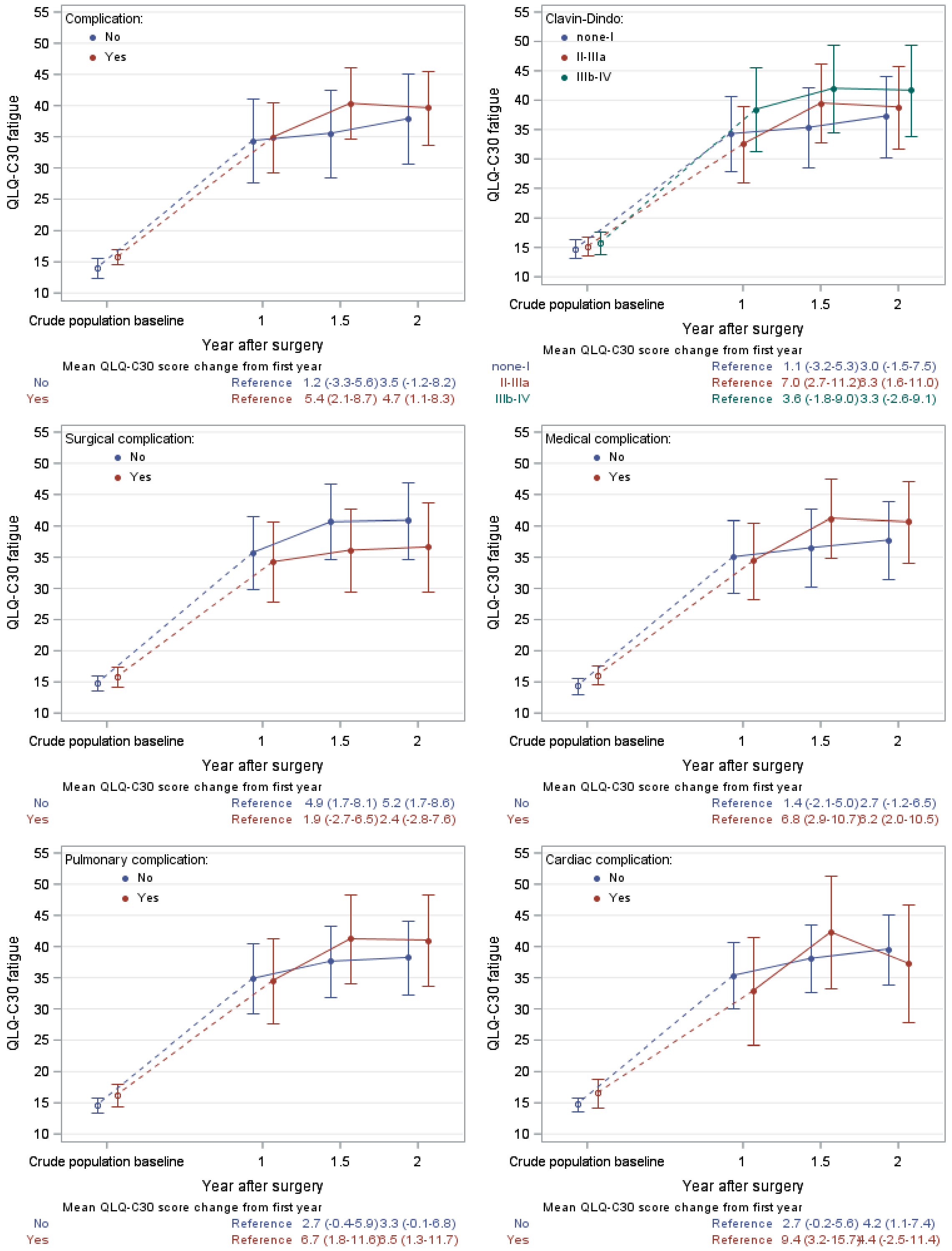

Figure A4. QLQ-C30 fatigue trajectories and mean score differences with 95% confidence intervals between time points by predefined complications (further adjusted for preoperative weight change)

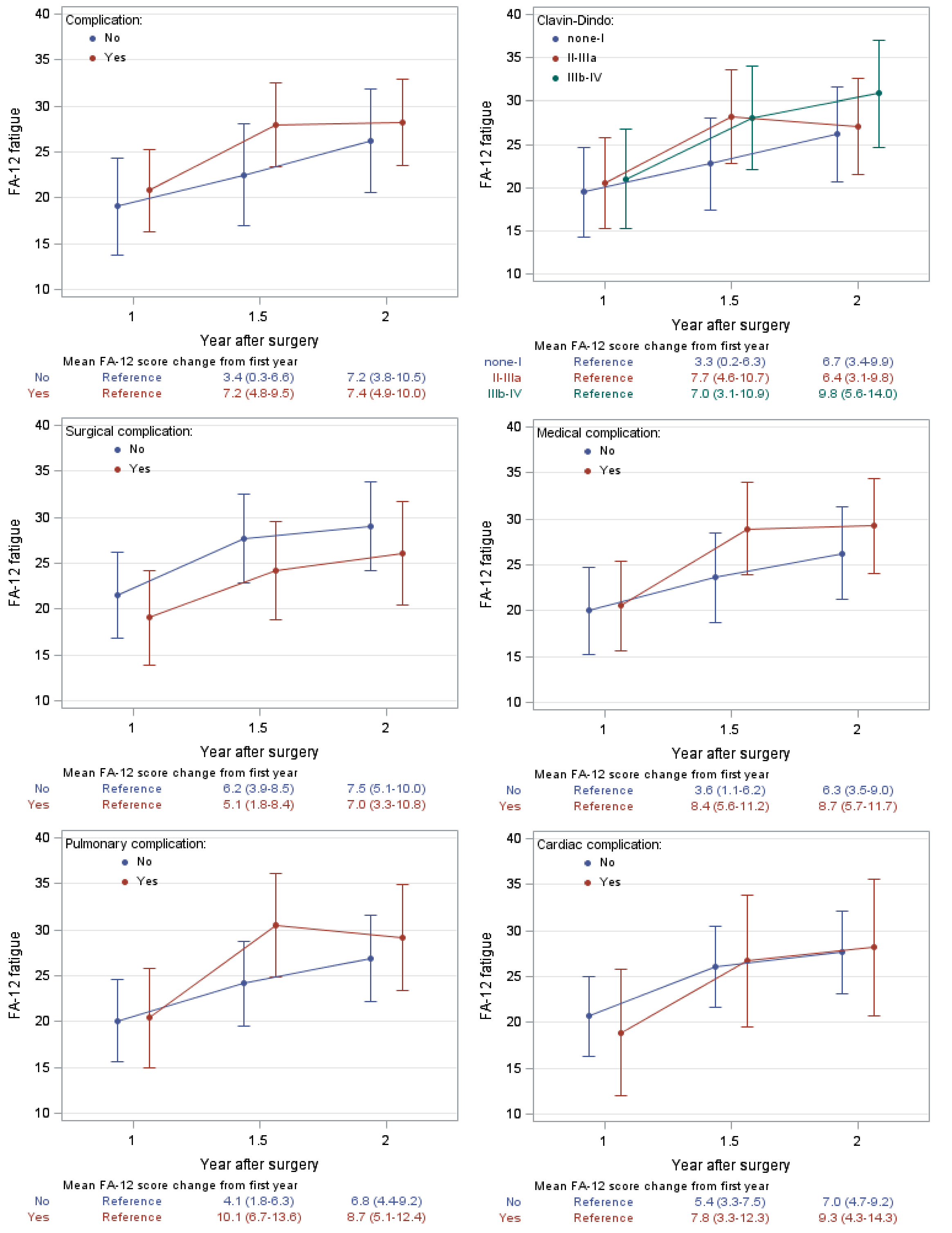

Figure A5. QLQ-FA12 fatigue trajectories and mean score differences with 95% confidence intervals between time points by predefined complication (further adjusted for preoperative weight change)

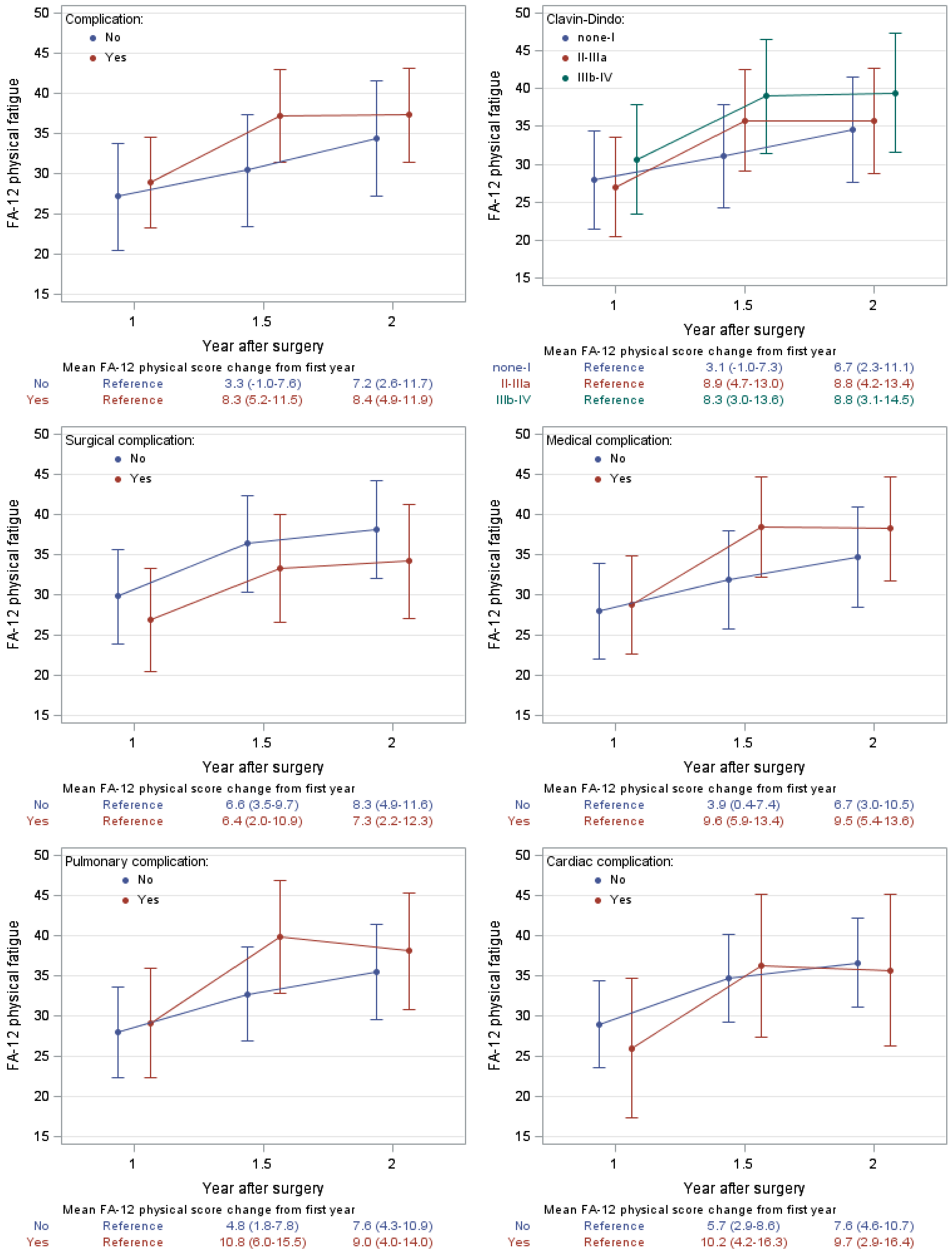

Figure A6. QLQ-FA12 physical fatigue trajectories and mean score differences with 95% confidence intervals between time points by predefined complication (further adjusted for preoperative weight change)

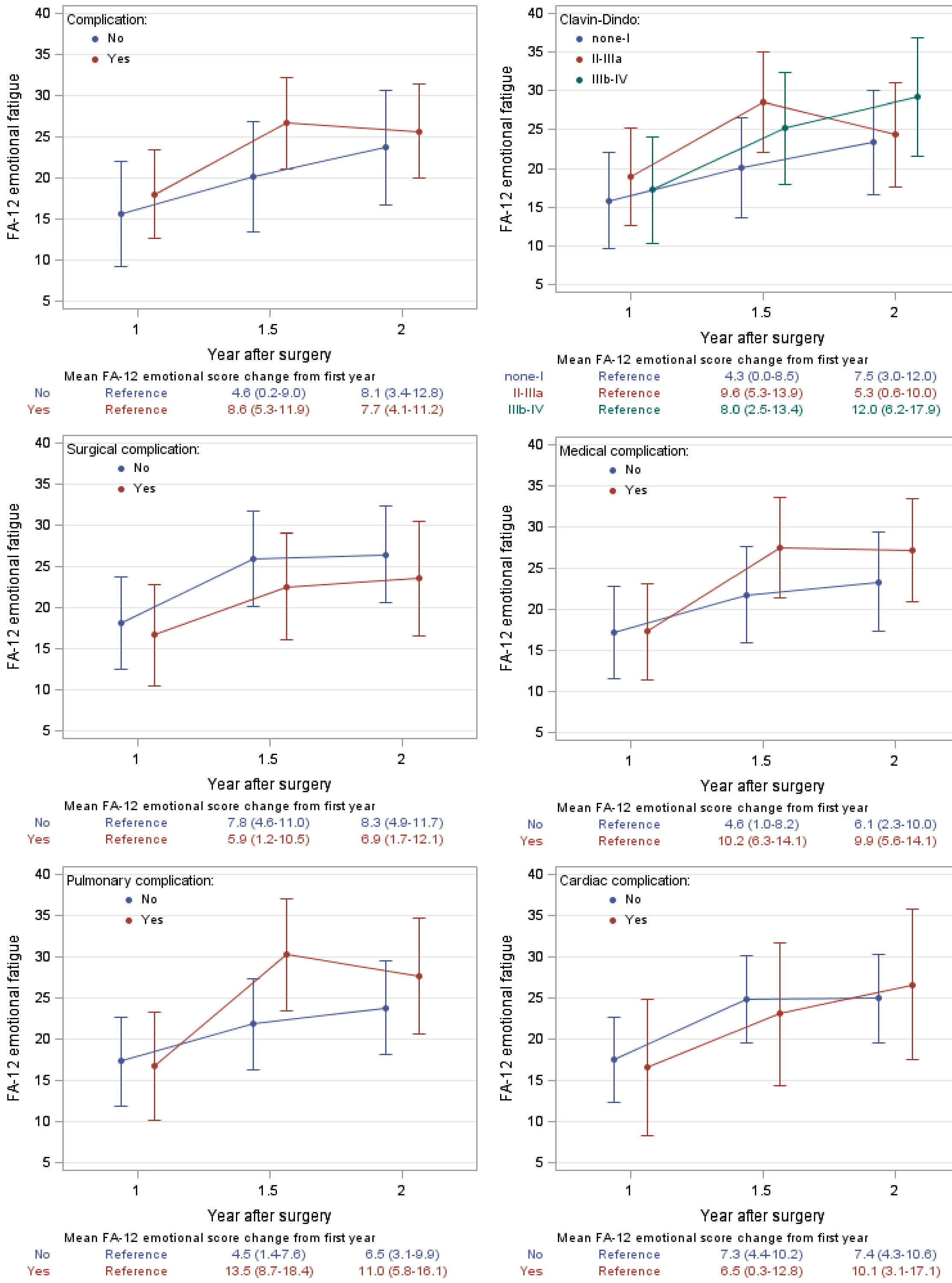

Figure A7. QLQ-FA12 emotional fatigue trajectories and mean score differences with 95% confidence intervals between time points by predefined complication (further adjusted for preoperative weight change)

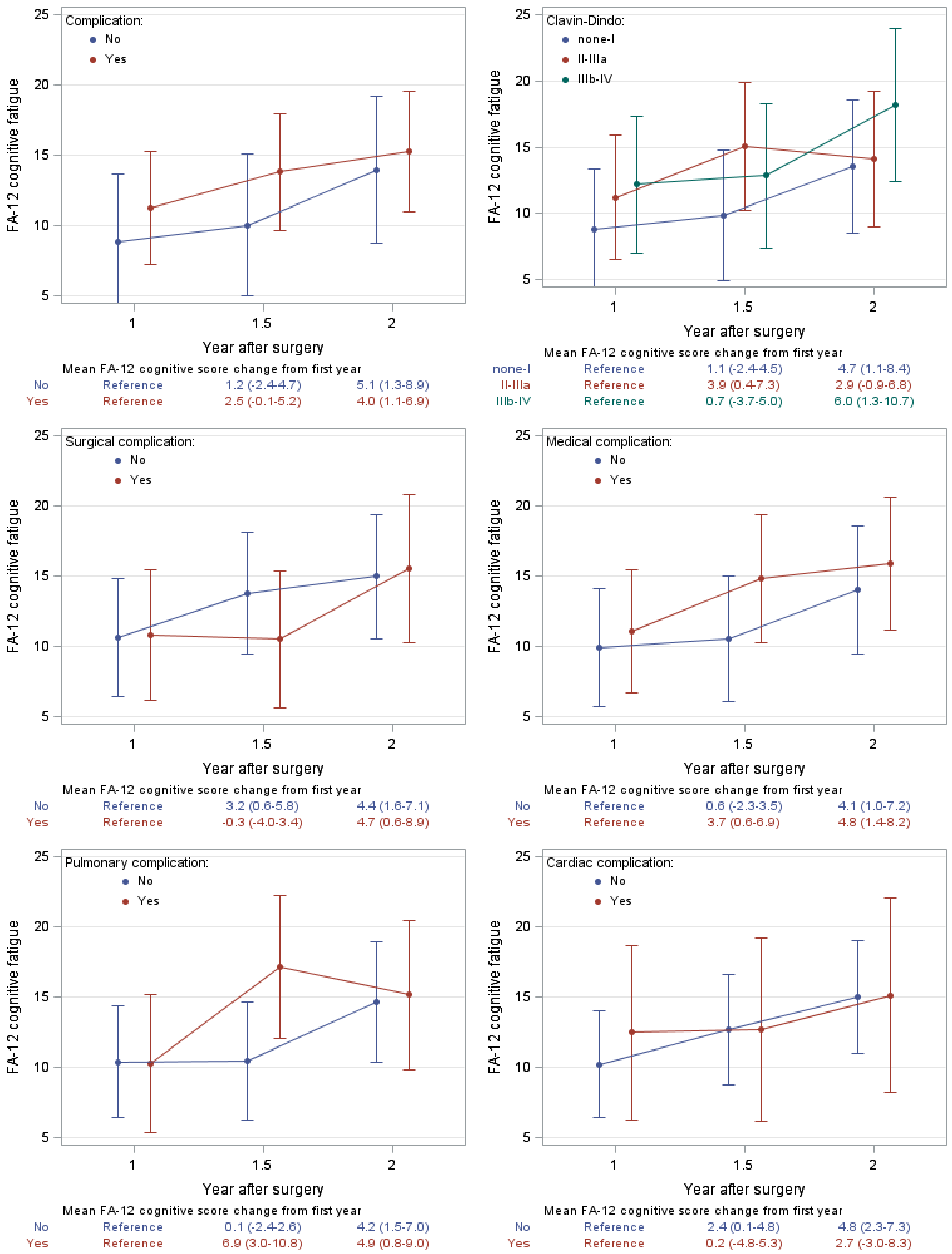

Figure A8. QLQ-FA12 cognitive fatigue trajectories and mean score differences with 95% confidence intervals between time points by predefined complication (further adjusted for preoperative weight change)

Table S4 Cause of death from the patients who died within 2 months of the response

Main cause of death	Tumor recurrence	N	Percent (%)
Unspecified location of malignant tumor in esophagus	Yes	20	60.6
Cardiac malignant tumor	Yes	2	6.1
Malignant tumor of the lower third of the esophagus	Yes	1	3.0
Unspecified location of malignant tumor of the stomach	Yes	1	3.0
Malignant tumor in Thyroid	Yes	1	3.0
Malignant tumor, unspecified primary location	Yes	1	3.0
Liver failure, unspecified	No	1	3.0
Intentional self-destructive action through shots from rifles, shotguns and heavier firearms-site	No	1	3.0
Missing	-	5	15.2
Total		33	100.0
